# Altered Gut Microbiota Related to Inflammatory Responses in Patients With Huntington’s Disease

**DOI:** 10.3389/fimmu.2020.603594

**Published:** 2021-02-19

**Authors:** Gang Du, Wei Dong, Qing Yang, Xueying Yu, Jinghong Ma, Weihong Gu, Yue Huang

**Affiliations:** ^1^ China National Clinical Research Center for Neurological Diseases, Beijing Tiantan Hospital, Capital Medical University, Beijing, China; ^2^ Centre for Neurological Diseases, Beijing Tiantan Hospital, Capital Medical University, Beijing, China; ^3^ Neurology Department, XuanWu Hospital, Capital Medical University, Beijing, China; ^4^ Neurology Department, China-Japan Friendship Hospital, Beijing, China; ^5^ School of Medical Sciences, Faculty of Medicine, University of New South Wales, Sydney, NSW, Australia

**Keywords:** Huntington’s disease, gut microbiota, 16S rDNA, cytokines, neuroinflammation

## Abstract

Emerging evidence indicates that gut dysbiosis may play a regulatory role in the onset and progression of Huntington’s disease (HD). However, any alterations in the fecal microbiome of HD patients and its relation to the host cytokine response remain unknown. The present study investigated alterations and host cytokine responses in patients with HD. We enrolled 33 HD patients and 33 sex- and age- matched healthy controls. Fecal microbiota communities were determined through 16S ribosomal DNA gene sequencing, from which we analyzed fecal microbial richness, evenness, structure, and differential abundance of individual taxa between HD patients and healthy controls. HD patients were evaluated for their clinical characteristics, and the relationships of fecal microbiota with these clinical characteristics were analyzed. Plasma concentrations of interferon gamma (IFN-γ), interleukin 1 beta (IL-1β), IL-2, IL-4, IL-6, IL-8, IL-10, IL-12p70, IL-13, and tumor necrosis factor alpha were measured by Meso Scale Discovery (MSD) assays, and relationships between microbiota and cytokine levels were analyzed in the HD group. HD patients showed increased α-diversity (richness), β-diversity (structure), and altered relative abundances of several taxa compared to those in healthy controls. HD-associated clinical characteristics correlated with the abundances of components of fecal microbiota at the genus level. Genus *Intestinimonas* was correlated with total functional capacity scores and IL-4 levels. Our present study also revealed that genus *Bilophila* were negatively correlated with proinflammatory IL-6 levels. Taken together, our present study represents the first to demonstrate alterations in fecal microbiota and inflammatory cytokine responses in HD patients. Further elucidation of interactions between microbial and host immune responses may help to better understand the pathogenesis of HD.

## Introduction

Genetic components are the dominant factors in the pathogeneses of monogenic neurodegenerative diseases. At present, increased attention has been focused on the role of the gut–brain axis and its related humoral response in the development of neurodegenerative diseases. However, the role of the gut–brain axis in monogenic neurodegenerative disease remains unclear.

Huntington’s disease (HD) is a monogenic, fully penetrant, progressive neurodegenerative disorder characterized by motor, cognitive, and psychiatric disturbances. HD is caused by the expansion of CAG trinucleotide repeats in exon 1 of the huntingtin (HTT) gene on chromosome 4, and HTT is widely expressed in the brain and in peripheral tissues such as skeletal muscles and the gut (1–4). The mutant huntingtin (mHTT) protein, which is expressed in the gastrointestinal (GI) tract, causes GI dysfunction, including impaired gut motility, diarrhea, and malabsorption of food (5). Malabsorption correlates with the amount of weight loss that is a hallmark of HD, both in patients with HD (6–9) and in several transgenic mouse models of HD (10). A recent study has suggested that gut dysbiosis may play a regulatory role in the onset age and progression of HD symptoms (11). In addition, mHTT is expressed in peripheral myeloid cells, including monocytes and macrophages (12, 13). Furthermore, monocytes from the blood of HD patients produce increased levels of cytokines ex vivo when stimulated (13, 14).

Gut microbiota may play a crucial role in the bidirectional gut–brain axis that affects brain activity under both physiological and pathological conditions (15). A growing number of studies suggests that gut microbiota exhibit physiological functions associated with neurodevelopment, brain function, and behavior (16–19). Maladaptive changes in the composition of gut microbiota, referred to as gut dysbiosis, have been linked to a number of gastrointestinal and metabolic diseases, including inflammatory bowel disease (IBD), obesity, and diabetes (20–22). In addition, gut dysbiosis has been implicated in various neurological and psychiatric diseases, such as autism spectrum disorder (ASD), major depression, amyotrophic lateral sclerosis (ALS), Parkinson’s disease (PD), and Alzheimer’s disease (AD) (23–28). Furthermore, R6/1 transgenic HD mice exhibit weight loss and gut-microbiota dysbiosis, the latter of which has been confirmed via 16S ribosomal RNA (rRNA) gene sequencing (11). Notably, alterations in circulating metabolites related to gut microbiota in HD patients and transgenic animals have suggested that changes in gut microbiota may occur before the onset of HD (29, 30).

However, there are currently no studies that have reported the composition of gut microbiota and related host cytokine responses in HD patients. Therefore, in the present study, we analyzed and compared the microbiota communities and peripheral cytokine levels of HD patients with those of healthy controls. Additionally, we analyzed the relationships between fecal microbiota and clinical characteristics in HD patients.

## Materials and Methods

### Study Subjects

All HD patients and controls in this study were recruited through a longitudinal study for aging and neurodegeneration project, which was conducted by the neurogenetic group at the China National Clinical Research Center for Neurological Diseases from September 2018 following international standard Enroll HD protocol (31), recommended by China HD Network (CHDN). All participating subjects signed informed consents prior to enrollment. This study was approved by the Research Ethics Committee at Beijing Tiantan Hospital, Capital Medical University, Beijing, China.

**Figure 1:**
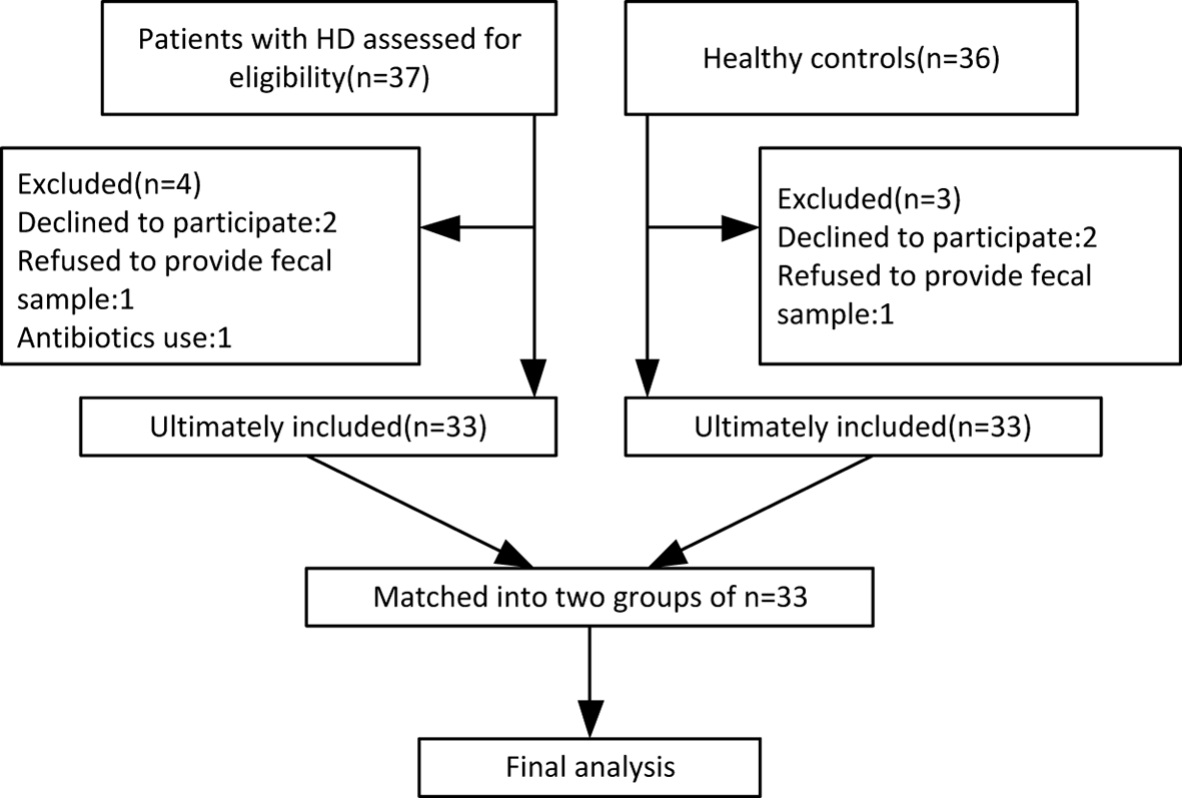
Flow chart of enrolled participants based on our exclusion and inclusion criteria.

By October 2019, 33 HD patients (24 manifest, 9 premanifest) and 33 sex- and age-matched healthy controls were recruited from 14 provinces across China. A study flow chart is presented in **Figure 1**. Each HD patient eligible for the present study received a diagnosis of HD according to a confirmed family history, positive genetic test, and motor disturbance as defined by the Unified HD Rating Scale (UHDRS) total motor score (TMS) diagnostic confidence score. The exclusion criteria were defined as severe chronic diseases such as diabetes, heart failure, liver cirrhosis, malignancy, hematological/autoimmune diseases, irritable bowel syndrome, and other movement disorders such as Wilson disease and chorea-acanthocytosis. The healthy controls were neurologically normal individuals and close relatives of the HD patients, such as neurologically normal spouse or parents, to ensure they shared similar environmental and dietary factors. If the patient’s offspring or siblings were included, genetic testing was conducted to confirm the offspring or siblings were not mutant HTT gene carriers. Individuals taking antibiotics or probiotic supplements within one month prior to sample collection were also excluded.

### Clinical Data Collection

Clinical data of subjects were collected *via* face-to-face interviews with HD researchers during the enrollment process. The HD researchers had already undertaken clinical-scales training under the instruction of the CHDN, which provided the Enroll HD clinical assessment package. The weight and height of each participant were measured, and body mass index (BMI) was calculated. HD clinical characteristics included disease duration and CAG repeats, as well as the motor section of the Unified Huntington’s Disease Rating Scale (UHDRS-M), the Functional Assessment Scale (FAS), Total Functional Capacity (TFC), Category Fluency Test (CFT), Symbol Digit Modalities Test (SDMT), Stroop Interference Test (SIT), Mini-Mental State Examination (MMSE), and Beck Depression Inventory II (BDI-II). To elaborate the quantitative clinical measures further, UHDRS-M ranges from 0 to 124 points with higher scores indicating more severe motor symptoms (32). FAS is a more detailed measure of functional capacity evaluation test and consists of 25 yes/no questions about specific functional abilities. The FAS examines tasks related to occupation (e.g., accustomed work, volunteer work), finances (e.g., cash transactions, financial management), activities of daily living (e.g., driving, hygiene), domestic chores (e.g., home maintenance, laundry), level of care (e.g., home or supervised care), and physical abilities (e.g., walking, getting out of bed, falls). FAS scores range from 0 to 25 with higher scores indicating greater functionality (33). The TFC provides a measure of broad functional capacity and consists of five global items that assess occupation, finances, domestic chores, activities of daily living, and care level. The scores on each item range from 0 to either 2 or 3. TFC total scores range from 0 to 13 with higher scores indicating greater functioning (33). The CFT requires the subject to name as many examples of the category ‘‘animal’’ as possible within 1 min (34). The SDMT requires participants to make as many symbol–number associations as possible within 90 s, and provides a measure of speed in information processing (35). SIT is a component of the Stroop Test that provides a measure of Executive Function (EF) including cognitive flexibility and resistance to interference. Scores reflect correct number in 45 s and higher scores indicate better performance (36). The MMSE provides a measure of general cognitive functioning (37). The BDI-II contains 21 items used to assess the intensity of the depression in clinically depressed or nondepressed patients. Each component is scored on a 4-point scale from 0 to 3 with higher scores indicating a more severe depressed mood (38).

### Sample Collection and DNA Extraction

Fecal samples were collected in tubes (SARSTEDT, Germany) with fecal preservation solution at home by the study participants according to our instructions and transported to our laboratory. Then, all samples were frozen immediately and stored at −80°C until further analysis. The following experiments were carried out commercially by Realbio Genomics Institute (Shanghai, China). DNA was extracted from each fecal sample *via* an improved protocol according to the manual of the QIAamp Fast DNA Stool Mini Kit (Qiagen, Germany). The concentration of genomic DNA in each fecal sample was quantified using a NanoDrop 2000 spectrophotometer (Thermo Scientific, MA, U.S.). The integrities and sizes of fecal DNA samples were assessed using 1% agarose gel electrophoresis.

Blood samples of 6 ml of venous blood were drawn from each participant at enrollment and were centrifuged (1,000 g for 15 min) within 1 h of collection. The plasma was aliquoted into cryotubes following centrifugation and stored at −80°C until further use for cytokine analysis.

### 16S rDNA Gene Amplicons and Sequencing

The V3–V4 regions of bacterial 16S rDNA genes were amplified by PCR (95°C for 3 min, followed by 30 cycles at 98°C for 20 s, 58°C for 15 s, and 72°C for 20 s, as well as a final extension at 72°C for 5 min) using universal primers (341F and 806R) linked with indices and sequencing adaptors. PCR amplification was performed in a 30-μL mixture containing 15 μL of 2 × KAPA Library Amplification ReadyMix, 1 μL of each primer (10 μM), 50 ng of template DNA, and ddH_2_O. Amplicons were extracted from 2% agarose gels and purified using an AxyPrep DNA Gel Extraction Kit (Axygen Biosciences, Union City, CA, U.S.) according to the manufacturer’s instructions. Purified amplicons were quantified using Qubit 2.0 (Invitrogen, U.S.). All quantified amplicons were sequenced using an Illumina NovaSeq PE250.

### Processing of Sequencing Data

Assembled tags, trimmed barcodes, and primers were further checked in terms of their rest lengths and average base qualities. 16S tags were restricted between 220–500 bp so that the average Phred score of bases was no worse than 20 (Q20) and no more than 3 ambiguous N. The copy number of tags was enumerated and redundancy of repeated tags was removed. Only tags with a frequency greater than 1, which tend to be more reliable, were clustered into operational taxonomic units (OTUs), each of which had a representative tag. OTUs were clustered based on 97% similarities using UPARSE and chimeric sequences were identified and removed using USEARCH (version 7.0.1090). Each representative tag was assigned to a taxa by the RDP Classifier (http://rdp.cme.msu.edu/) against the RDP database (http://rdp.cme.msu.edu/) using a confidence threshold of 0.8. The α-diversity and β-diversity indices were calculated based on the rarefied OTU counts *via* Qiime v1.9.1. Specifically, α-diversity represents an analysis of diversity in a single sample reflected by parameters including good coverage, Chao 1, PD whole tree, Shannon index, and Simpson index, using Qiime (39). The Wilcox test in R software was used to compare each α-diversity index. β-diversity is used as a measure of the microbiota structure between groups. The results of both the weighted and unweighted Unifrac distance matrices were plotted in the principal coordinate analysis (PCoA), and Adonis was performed using R software. Microbial features used to distinguish fecal microbiotas specific to HD were identified using the linear discriminant analysis (LDA) effect size (LEfSe) method (http://huttenhower.sph.harvard.edu/lefse/) with an alpha cutoff of 0.05 and an effect-size cutoff of 2.0.

### Measurement of Plasma Cytokine Levels

The V-PLEX proinflammatory Panel 1 human kit [Meso Scale Discovery (MSD)] was used to measure plasma concentrations of IFN-γ, IL-10, IL-12p70, IL-13, IL-1β, IL-2, IL-4, IL-6, IL-8, and TNF-α according to the manufacturer protocol.

### Statistical Analysis

The SPSS (ver. 21.0, SPSS Inc., Chicago, IL, USA) and R software (ver. 3.5.1, the R Project for Statistical Computing) were used for statistical analysis. Differences between groups were determined *via* Student’s t-test and Pearson’s Chi-squared tests for quantitative and categorical variables, respectively. Variables that violated the assumptions of normality were compared *via* nonparametric Mann-Whitney U tests. Correlations among components of fecal microbiota with clinical parameters and cytokines were determined *via* Spearman correlation analysis. Statistical significance was set at *p* < 0.05.

## Results

### Demographic and Clinical Characteristics of HD Patients and Healthy Controls

The demographic characteristics of HD patients and healthy controls in this study are presented in **Table 1**. There were no significant differences between the HD and controls in terms of age or gender (**Table 1**), but the age at assessment in the premanifest HD is much younger compared to the manifested HD (**Supplementary Table 1**). The median duration of HD patients at enrollment was 4 years, and the median number of CAG repetitions was 42. HD patients has a significantly lower BMI compared to that of healthy controls (**Table 1**).

**Table 1:** Demographics and clinical characteristics of HD patients and healthy controls.

	HD group	Healthy control group	*p* value
N	33	33	NA
M:F	15:18	15:18	NA
Age (y/o)	42.6 (12.7)^a^	48.0 (13.5)^a^	0.0967^*^
BMI (kg/m^2^)	21.3 (3.7)^a^	24.0 (3.6)^a^	0.003^*^
HD duration(y)	4.0 (7.0)^b^	NA	NA
CAG repeat number	42.0 (3.5)^b^	NA	NA
UHDRS-M	35.0 (70.0)^b^	NA	NA
FAS scores	21.0 (11.0)^b^	NA	NA
TFC scores	12.0 (10.0)^b^	NA	NA
SIT scores	18.0 (16.0)^b^	–	NA
SDMT scores	21.0 (25.0)^b^	–	NA
CFT scores	11.0 (11.0)^b^	–	NA
BDI-II scores	6.0 (15.0)^b^	–	NA
MMSE scores	28.0 (5.0)^b^	–	NA

*Unpaired t-test; the data are presented as the mean (SD)^a^ or median (IQR)^b^; HD, Huntington’s disease; NA, not applicable; SD, standard deviation; IQR, interquartile range; M:F, male: female; y, years; y/o, years old; BMI, body mass index; UHDRS-M, motor section of Unified Huntington’s Disease Rating Scale; FAS, Functional Assessment Scale; TFC, Total Functional Capacity; SIT, Stroop Interference Test; SDMT, Symbol Digit Modalities Test; CFT, Category Fluency Test; BDI-II, Beck Depression Inventory II; MMSE, Mini-Mental State Examination.

### Alpha and Beta Diversity Between HD Patients and Healthy Controls

**Figure 2:**
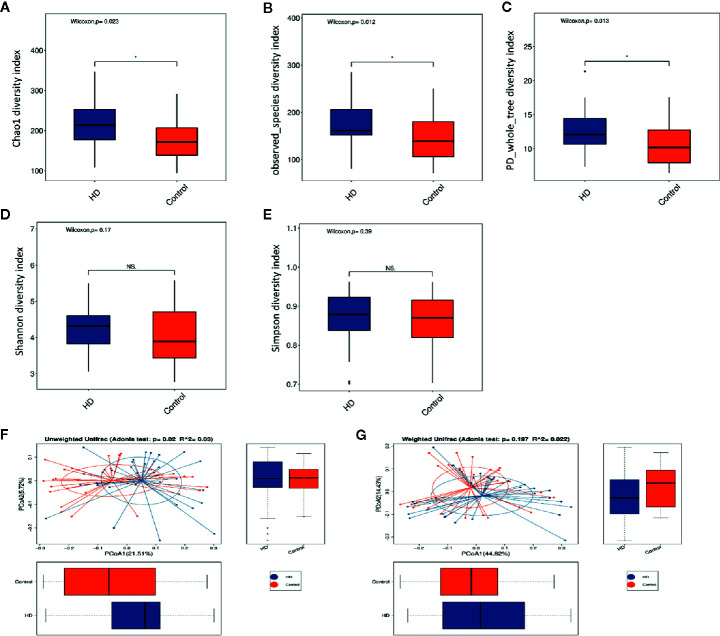
Comparisons of α-diversity indexes (richness and evenness) of the fecal microbiota between HD and healthy controls. **(A–C)**, Comparisons of richness (Chao 1 index, observed species index, and PD whole tree index) between HD and healthy controls. **(D, E)**, Comparisons of evenness (Shannon index and Simpson index) between HD patients and healthy controls. *p < 0.05; “NS.” means no significant difference. β-diversity analyses using Adonis and unweighted **(F)** and weighted **(G)** PCoA based on the distance matrix of UniFrac dissimilarity of the fecal microbiota in HD patients and healthy controls.

The dilution curves of α-diversity indices were plotted to demonstrate that the sample size in this study is adequate for valid analysis (**Supplementary Figure 1**). The Chao 1, observed species, and PD whole tree of the HD group were significantly higher than those of the healthy control group, whereas there were no significant differences between these two groups in terms of the Shannon and Simpson index (**Figures 2A–E**). These results indicate that the richness of the gut microbiota in the HD group was significantly higher than that of the healthy control group. Significant differences were also found in β-diversity based on the unweighted (qualitative, Adonis R^2^ = 0.03, *p* = 0.02) but not the weighted (quantitative, Adonis R^2^ = 0.022, *p* = 0.197). UniFrac between HD and healthy control groups (**Figures 2F, G**), indicating that the fecal microbial structure, but not the abundance, in the HD group was significantly different from that of the healthy control group.

### Compositions of Microbial Taxa at Multiple Phylogenetic Ranks Between HD Patients and Healthy Controls

**Figure 3:**
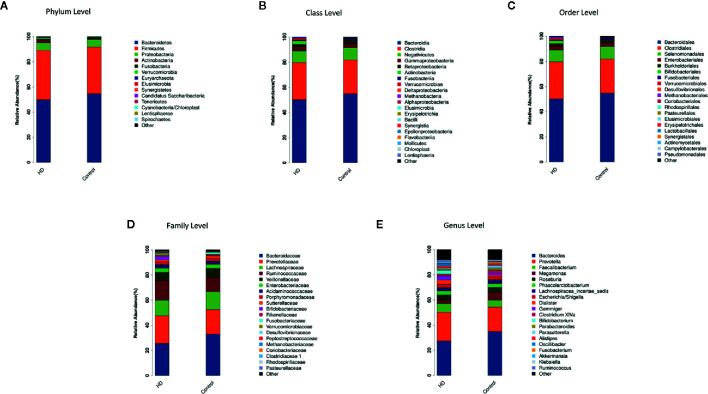
Compositions and relative abundances of taxa at multiple phylogenetic ranks based on 16S rDNA sequences in HD patients and healthy controls. The compositions and relative abundances of major taxa in HD and controls are compared at level of phylum **(A)**, class **(B)**, order **(C)**, family **(D)** and genus **(E)**.

The top-20 taxa at multiple phylogenetic ranks were analyzed. For example, at the genus level, *Bacteroides* and *Prevotella* constituted two common dominant genera in both the HD group and healthy control group (*Bacteroides*: 27.36 vs. 34.93%, respectively; *Prevotella*: 22.80 vs. 19.39%), which accounted for 50.16 and 54.32% of the total sequencing number. In addition, the average ratios of *Bacteroides* and *Prevotella* between the HD patients and healthy controls were 0.78 and 1.18, respectively (**Figure 3**). The microbial species profiling histogram of the HD cohort were also demonstrated according to the disease duration (**Supplementary Figures 2**–**6**).

### Altered Microbiota Between HD Patients and Healthy Controls

**Figure 4:**
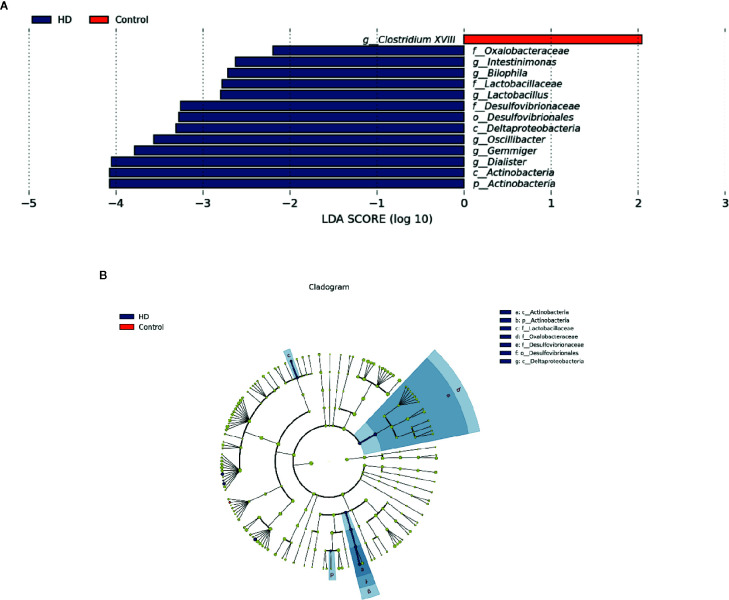
Fecal microbiota differences between HD patients and healthy controls detected by LEfSe analysis. **(A)** Linear discriminant analysis (LDA) effect size (LEfSe) analysis showing significant bacterial differences in fecal microbiota between the HD patients and healthy controls. The LDA scores (log10) > 2 and *p* < 0.05 are listed. **(B)** A cladogram showing the taxonomic structure and relative abundances of the identified taxa. The size of each dot is proportional to the relative abundance of each taxon (p, phylum; c, class; o, order; f, family; g, genus).

To determine the significantly increased bacteria in the HD group or healthy control group, supervised comparisons *via* LEfSE (LDA > 2.0) were performed. This LEfSe analysis revealed many significant differences in the fecal microbiota between the HD group and healthy control group. Specifically, the following relative abundances in the HD group were significantly higher than those in the healthy control group: *Actinobacteria* at the phylum level; *Deltaproteobacteria* and *Actinobacteria* at the class level; *Desulfovibrionales* at the order level; *Oxalobacteraceae*, *Lactobacillaceae*, and *Desulfovibrionaceae* at the family level; and *Intestinimonas*, *Bilophila*, *Lactobacillus*, *Oscillibacter*, *Gemmiger*, and *Dialister* at the genus level. In contrast, *Clostridium XVIII* at genus level was significantly higher in the healthy control group compared to that in the HD group (**Figure 4**).

### Relationships Between Fecal Microbiota and HD Clinical Characteristics

**Figure 5:**
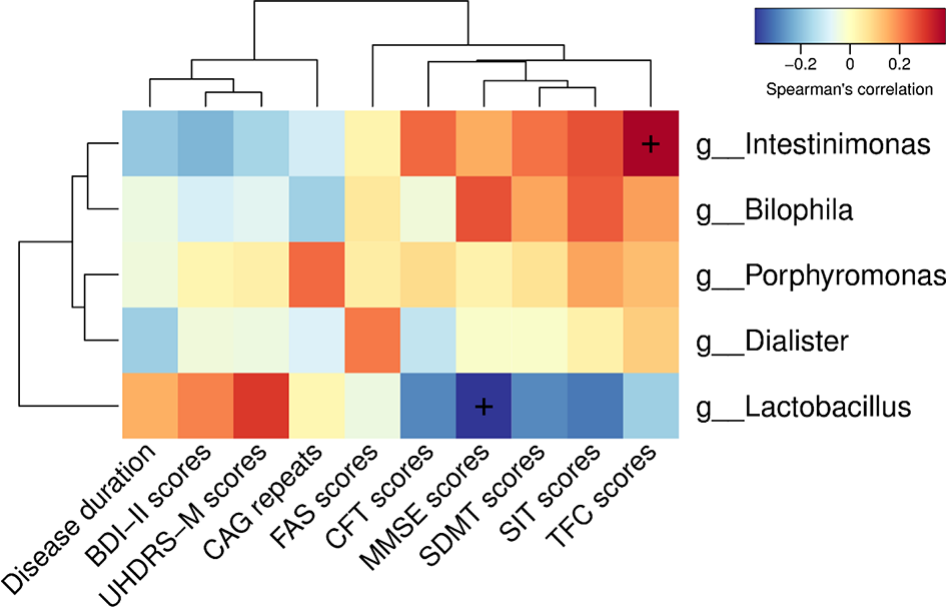
Correlations among clinical characteristics and gut-microbiota genera obtained by LEfSe analysis. Correlation heatmaps showing the relationships among clinical characteristics and gut-microbiota genera. Each row in the heatmap represents a genus. Each column represents a clinical characteristic. The color temperature encodes the Spearman correlation coefficient (ρ). The sample size was n = 33. The “+” symbol within a cell indicates a *p* < 0.05. Abbreviations are as follows: BDI-II scores, Beck Depression Inventory II scores; UHDRS-M, motor section of the Unified Huntington’s Disease Rating Scale scores; FAS scores, Functional Assessment Scale scores; CFT scores, Category Fluency Test scores; MMSE scores, Mini-Mental State Examination scores; SDMT scores, Symbol Digit Modalities Test scores; SIT scores, Stroop Interference Test scores; TFC scores, Total Functional Capacity scores.

To determine the relationship between clinical characteristics and gut microbiota in HD patients, Spearman correlation analysis was performed to evaluate correlations among clinical characteristics and gut-microbiota genera obtained by LEfSe analysis. Our analysis revealed two correlations between fecal microbiota at the genus level with specific clinical scores, namely *Intestinimonas* with TFC scores and *Lactobacillus* with MMSE scores (**Figure 5**).

### Cytokine Responses in HD Patients With Alterations in Fecal Microbiota

**Figure 6:**
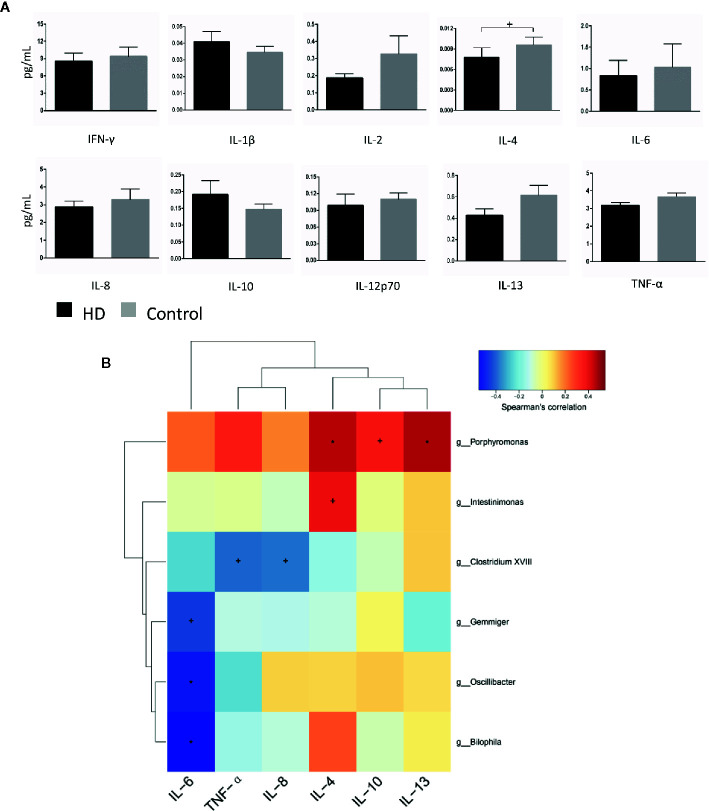
Cytokine profiles in HD patients and healthy controls, as well as correlations between cytokines and gut-microbiota genera obtained by LEfSe analysis. **(A)** Plasma concentrations of interferon-gamma (IFN-γ), interleukin-1β (IL-1β), IL-2, IL-4, IL-6, IL-8, IL-10, IL-12p70, IL-13, and tumor necrosis factor alpha (TNF-α). **(B)** Correlation heatmaps showing the relationships among cytokines and gut-microbiota genera. Each row in the heatmap represents a genus. Each column represents a cytokine. The color temperature encodes the Spearman correlation coefficient (ρ). The sample size was n = 33. Statistical analyses were performed by Mann–Whitney tests **(A)** and Spearman’s tests **(B)**. The error bars indicate the standard error of mean (SEM). HD denotes Huntington’s disease. ^+^ *p* < 0.05, ^*^ *p <* 0.01.

To evaluate plasma cytokine profiles from HD patients and healthy controls, we quantified the plasma concentrations of interferon gamma (IFN-γ), interleukin 1 beta (IL-1β), IL-2, IL-4, IL-6, IL-8, IL-10, IL-12p70, IL-13, and tumor necrosis factor alpha (TNF-α). IL-4 plasma concentrations, characteristic of responses from T-helper-2 cells, was significantly lower (*p* = 0.03) in plasma samples from HD patients (0.008 ± 0.001 pg/mL) than in samples from healthy controls (0.009 ± 0.001 pg/mL). In contrast, there were no significant differences (*p* > 0.05) in the plasma concentrations of IFN-γ, IL-1β, IL-2, IL-6, IL-8, IL-10, IL-12p70, IL-13, or TNF-α in HD patients (**Figure 6A**).

Finally, to identify correlations between components of fecal microbiota and cytokines, we examined correlations between systemic levels of cytokines and relative abundances of fecal microbiota in the HD group. We found correlations between fecal microbiota and cytokines (e.g., *Intestinimonas* with plasma IL-4 levels (*p* = 0.028, ρ = 0.382), *Bilophila* with plasma IL-6 levels (*p* = 0.001, ρ = −0.544) (**Figure 6B**).

## Discussion

The present study provides the evidence for gut dysbiosis in human patients with HD, providing clinical relevance to a previous study that reported gut dysbiosis in a transgenic mouse model of HD (11). We found that *Intestinimonas* and *Bilophila* correlated with concentrations of IL-4 and IL-6, respectively, in HD patients, suggesting the occurrence of a systemic chronic inflammatory status associated with altered gut microbiota.

In our present study, we used 16S rDNA gene sequencing on DNA isolated from fecal samples to systematically analyze characteristics of gut microbiota between HD patients and healthy controls. We observed that α-diversity (richness) in HD patients was significantly higher than that of healthy controls, as was *β*-diversity (structure), consistent with previous results from a mouse model of HD (11). However, in a recent study of the gut microbiota of HD patients (40), the α-diversity of HD patients was lower than that of healthy controls. The disparity is likely due to different ethnic origins, geography, host, genetics, age and other factors (41). Previous study showed an increase in gut microbiota richness was detected in autistic children (42), and it shared great similarities in the altered microbiota species with HD. Given HD was also considered as a neurodevelopmental disorder similar to autism (43), the greater bacterial diversity is potentially beneficial to the development of central nervous system (CNS) function, which remains subject to debate.

Furthermore, we analyzed the top-20 taxa at multiple phylogenetic ranks. At the genus level, the average ratios of *Bacteroides* and *Prevotella* between groups HD and healthy control were 0.78 and 1.18. The significant bacterial differences identified by LEfSe (LDA > 2.0) showed that *Clostridium XVIII* at the genus level was significantly higher in the healthy control group compared to that in the HD group, whereas the relative abundances of the following were higher in the HD group compared to those in the healthy control group: *Actinobacteria* at the phylum level; *Deltaproteobacteria* and *Actinobacteria* at the class level; *Desulfovibrionales* at the order level; *Oxalobacteraceae*, *Lactobacillaceae*, and *Desulfovibrionaceae* at the family level; and *Intestinimonas*, *Bilophila*, *Lactobacillus*, *Oscillibacter*, *Gemmiger*, and *Dialister* at the genus level. These results indicate that particular gut microbiota components are associated with HD. The increased abundance of *Actinobacteria* at the phylum level in the HD group compared to that in the healthy control group in our present study is similar to previous findings reported in patients with IBD (44), type-2 diabetes mellitus (T2DM) (45), AD (28), ASD (46), and major depressive disorder (47, 48). The increased abundance of *Actinobacteria* at the class level in the HD patients compared to that in the healthy controls in this study is consistent with previous studies in AD and ASD patients (28, 46). Family level analysis in our present study revealed significant increases in the abundance of *Desulfovibrionaceae* in the fecal samples of HD patients compared to those from healthy controls, whereas a previous study found that the abundance of *Desulfovibrionaceae* was increased in the feces of IBD patients (49), which is likely a breaker of the intestinal mucosal barrier that induces IBD (50).

The abundance of the *Intestinimonas* genus was higher in HD patients than in healthy controls and correlated with TFC scores. A previous study has shown that *Intestinimonas*, which is correlated with propionic/butyric acid, plays a key role in anti-inflammation (51, 52). In the present study, we observed a positive correlation between *Intestinimonas* and plasma concentrations of IL-4, an anti-inflammatory cytokine mainly produced by T-helper-2 cells (53). Since butyrate has the ability to regulate T cell differentiation (54), it suggests that *Intestinimonas* may confer efficacious anti-inflammatory effects on systemic inflammatory responses in HD patients. However, future studies are needed to reveal the interactions between changes in symbiotic gut microbiota and the immune reactions in HD pathogenesis.

*Bilophila* contains only species of *B. wadsworthia*, and a higher abundance of *B. wadsworthia* induces systemic inflammatory responses in specific pathogen-free (SPF) mice (55). In addition, *B. wadsworthia* plays an important role in the development of IBD-like colitis in *IL-10* ^-/-^ mice (56). Additionally, *B. wadsworthia* has been found to be positively associated with proinflammatory cytokines, such as IL-6 (57) and IL-1β (58). A previous study found that *Bilophila* was significantly negatively correlated (*p* < 0.05) with IL-6 mRNA levels in a T2DM rat model treated with stachyose (59). Interestingly, our present study also revealed that *Bilophila* were negatively correlated with proinflammatory IL-6 levels, suggesting that *Bilophila* may play an anti-inflammatory role in systemic inflammatory responses in HD patients. That said, further research is needed to determine the precise relationships between gut microbiota compositions and immune reactions in HD patients.

*Lactobacillus* has the ability to ferment a series of carbon sources, primarily to lactic acid, and is widely recognized as a source of probiotics (60). Several recent studies have found that *Lactobacillus* is more abundant in patients with T2DM (61), ASD (62, 63), IBD (64), and rheumatoid arthritis (65, 66), which is consistent with our present findings in HD patients. Interestingly, we found that the abundance of *Lactobacillus* was negatively correlated with MMSE scores, although we did not find any correlation between *Lactobacillus* and levels of inflammatory cytokines. These findings suggest that if the abundance of *Lactobacillus* adversely impacts MMSE scores, and consequently cognitive function, it likely does so *via* a non-cytokine-induced mechanism.

In the present study, we found that the abundance of *Clostridium XVIII* was significantly decreased in the HD group compared to that in healthy controls, indicating higher fecal water content in the HD group, similar to findings in HD mice (11). Strati et al. observed that *Clostridium XVIII* was significantly more abundant in constipated autistic subjects compared to that in non-constipated autistic subjects, and this increased abundance was associated with clinical manifestations of GI problems related to alterations in gut microbiota (62).

There are several limitations of this study. Although we managed to recruit HD and control pairs from the same family and at the same geographic location across China, the sample size is minimal and the nature of the present study is exploratory. Although this study was controlled for factors affecting the gut microbiota, such as age, gender, diet, and region, physical activity, smoking, alcohol, drugs and other factors should be fully considered in the further study. Further studies with larger sample sizes comprised of independent cohorts originating from a different population are required to consolidate our findings of different microbial species between HD patients and healthy controls, as well as the microbial species associated with clinical symptoms. In addition, more advanced technology such as shotgun metagenome analysis, and more advanced association analysis platform such as HALLA (67) can be used in the future to provide more detailed information regarding any implicated microbiota in future studies to more comprehensively understand fecal microbiota composition in HD patients.

In summary, the present study first elucidates that gut microbiota are altered in HD patients and are correlated with specific clinical characteristics. Furthermore, we demonstrated that fecal microbiota was related to specific cytokine levels. Revealing the precise interactions between microbial and host immune responses may help to better understand the pathogenesis of HD.

## Data Availability Statement

The datasets presented in this study can be found in online repositories. The names of the repository/repositories and accession number can be found below: National Center for Biotechnology Information (NCBI) BioProject database with project number PRJNA667318.

## Ethics Statement

The studies involving human participants were reviewed and approved by Ethics Committee, Beijing Tiantan Hospital, Capital Medical University, Beijing, China. The patients/participants provided their written informed consent to participate in this study.

## Author Contributions

Clinical analyses and manuscript writing: GD. Participants recruitment: GD, WD, XY, WG, JM, and YH. Samples preparation: QY and WD. Experimental design and critical revision: YH. All authors contributed to the article and approved the submitted version.

## Funding

This work was supported by Beijing Tiantan Hospital, Capital Medical University for Neurogenetics Studies (YH) and Beijing Postdoctoral Research Foundation (GD).

## Conflict of Interest

The authors declare that the research was conducted in the absence of any commercial or financial relationships that could be construed as a potential conflict of interest.

## Supplementary Material

Supplementary Figure 1 Dilution curves of α-diversity index. As the amount of sequencing increased, more species were found, and no new OTU was found by increasing the number of sampling strips until the species was saturated.

Supplementary Figure 2 Species profiling histogram of the HD samples according to HD duration at phylum classification level.

Supplementary Figure 3 Species profiling histogram of the HD samples according to HD duration at class classification level.

Supplementary Figure 4 Species profiling histogram of the HD samples according to HD duration at order classification level.

Supplementary Figure 5 Species profiling histogram of the HD samples according to HD duration at family classification level.

Supplementary Figure 6 Species profiling histogram of the HD samples according to HD duration at genus classification level.

## References

[1] Moffitt H McPhail GD Woodman B Hobbs C Bates GP. Formation of polyglutamine inclusions in a wide range of non-CNS tissues in the HdhQ150 knock-in mouse model of Huntington’s disease. PloS One (2009) 4:e8025. 10.1371/journal.pone.0008025 19956633 PMC2778556

[2] Sathasivam K Hobbs C Turmaine M Mangiarini L Mahal A Bertaux F. Formation of polyglutamine inclusions in non-CNS tissue. Hum Mol Genet (1999) 8:813–22. 10.1093/hmg/8.5.813 10196370

[3] Sharp AH Loev SJ Schilling G Li SH Li XJ Bao J. Widespread expression of Huntington’s disease gene (IT15) protein product. Neuron (1995) 14:1065–74. 10.1016/0896-6273(95)90345-3 7748554

[4] Listed N. A novel gene containing a trinucleotide repeat that is expanded and unstable on Huntington’s disease chromosomes. Cell (1993) 72:971–83. 10.1016/0092-8674(93)90585-E 8458085

[5] van der Burg JM Winqvist A Aziz NA Maat-Schieman ML Roos RA Bates GP. Gastrointestinal dysfunction contributes to weight loss in Huntington’s disease mice. Neurobiol Dis (2011) 44:1–8. 10.1016/j.nbd.2011.05.006 21624468

[6] Djoussé L Knowlton B Cupples LA Marder K Shoulson I Myers RH. Weight loss in early stage of Huntington’s disease. Neurology (2002) 59:1325–30. 10.1212/01.wnl.0000031791.10922.cf 12427878

[7] Farrer LA Meaney FJ. An anthropometric assessment of Huntington’s disease patients and families. Am J Phys Anthropol (1985) 67:185–94. 10.1002/ajpa.1330670304 2932916

[8] Sanberg PR Fibiger HC Mark RF. Body weight and dietary factors in Huntington’s disease patients compared with matched controls. Med J Aust (1981) 1:407–9. 10.5694/j.1326-5377.1981.tb135681.x 6454826

[9] Trejo A Tarrats RM Alonso ME Boll MC Ochoa A Velásquez L. Assessment of the nutrition status of patients with Huntington’s disease. Nutr (Burbank Los Angeles County Calif) (2004) 20:192–6. 10.1016/j.nut.2003.10.007 14962685

[10] Menalled LB Chesselet MF. Mouse models of Huntington’s disease. Trends Pharmacol Sci (2002) 23:32–9. 10.1016/s0165-6147(00)01884-8 11804649

[11] Kong G Cao KL Judd LM Li S Renoir T Hannan AJ. Microbiome profiling reveals gut dysbiosis in a transgenic mouse model of Huntington’s disease. Neurobiol Dis (2020) 135:104268. 10.1016/j.nbd.2018.09.001 30194046

[12] Weiss A Trager U Wild EJ Grueninger S Farmer R Landles C. Mutant huntingtin fragmentation in immune cells tracks Huntington’s disease progression. J Clin Invest (2012) 122:3731–6. 10.1172/JCI64565 PMC3461928 22996692

[13] Bjorkqvist M Wild EJ Thiele J Silvestroni A Andre R Lahiri N. A novel pathogenic pathway of immune activation detectable before clinical onset in Huntington’s disease. J Exp Med (2008) 205:1869–77. 10.1084/jem.20080178 PMC2525598 18625748

[14] Trager U Andre R Lahiri N Magnusson-Lind A Weiss A Grueninger S. HTT-lowering reverses Huntington’s disease immune dysfunction caused by NFkappaB pathway dysregulation. Brain (2014) 137:819–33. 10.1093/brain/awt355 PMC3983408 24459107

[15] Wang Y Kasper LH. The role of microbiome in central nervous system disorders. Brain Behav Immun (2014) 38:1–12. 10.1016/j.bbi.2013.12.015 24370461 PMC4062078

[16] Clarke G Grenham S Scully P Fitzgerald P Moloney RD Shanahan F. The microbiome-gut-brain axis during early life regulates the hippocampal serotonergic system in a sex-dependent manner. Mol Psychiatry (2013) 18:666–73. 10.1038/mp.2012.77 22688187

[17] Hoban AE Stilling RM Moloney G Shanahan F Dinan TG Clarke G. The microbiome regulates amygdala-dependent fear recall. Mol Psychiatry (2018) 23:1134–44. 10.1038/mp.2017.100 PMC5984090 28507320

[18] Ogbonnaya ES Clarke G Shanahan F Dinan TG Cryan JF O’Leary OF. Adult Hippocampal Neurogenesis Is Regulated by the Microbiome. Biol Psychiatry (2015) 78:e7–9. 10.1016/j.biopsych.2014.12.023 25700599

[19] Stilling RM Ryan FJ Hoban AE Shanahan F Clarke G Claesson MJ. Microbes & neurodevelopment–Absence of microbiota during early life increases activity-related transcriptional pathways in the amygdala. Brain Behav Immun (2015) 50:209–20. 10.1016/j.bbi.2015.07.009 26184083

[20] Greenblum S Turnbaugh PJ Borenstein E. Metagenomic systems biology of the human gut microbiome reveals topological shifts associated with obesity and inflammatory bowel disease. Proc Natl Acad Sci U States America (2012) 109:594–9. 10.1073/pnas.1116053109 PMC3258644 22184244

[21] Qin J Li Y Cai Z Li S Zhu J Zhang F. A metagenome-wide association study of gut microbiota in type 2 diabetes. Nature (2012) 490:55–60. 10.1038/nature11450 23023125

[22] Turnbaugh PJ Ley RE Mahowald MA Magrini V Mardis ER Gordon JI. An obesity-associated gut microbiome with increased capacity for energy harvest. Nature (2006) 444:1027–31. 10.1038/nature05414 17183312

[23] Ma B Liang J Dai M Wang J Luo J Zhang Z. Altered Gut Microbiota in Chinese Children With Autism Spectrum Disorders. Front Cell Infect Microbiol (2019) 9:40:40. 10.3389/fcimb.2019.00040 30895172 PMC6414714

[24] Jiang H Ling Z Zhang Y Mao H Ma Z Yin Y. Altered fecal microbiota composition in patients with major depressive disorder. Brain Behav Immun (2015) 48:186–94. 10.1016/j.bbi.2015.03.016 25882912

[25] Brenner D Hiergeist A Adis C Mayer B Gessner A Ludolph AC. The fecal microbiome of ALS patients. Neurobiol Aging (2018) 61:132–7. 10.1016/j.neurobiolaging.2017.09.023 29065369

[26] Qian Y Yang X Xu S Wu C Song Y Qin N. Alteration of the fecal microbiota in Chinese patients with Parkinson’s disease. Brain Behav Immun (2018) 70:194–202. 10.1016/j.bbi.2018.02.016 29501802

[27] Wallen ZD Appah M Dean MN Sesler CL Factor SA Molho E. Characterizing dysbiosis of gut microbiome in PD: evidence for overabundance of opportunistic pathogens. NPJ Parkinson’s Dis (2020) 6:11. 10.1038/s41531-020-0112-6 32566740 PMC7293233

[28] Zhuang ZQ Shen LL Li WW Fu X Zeng F Gui L. Gut Microbiota is Altered in Patients with Alzheimer’s Disease. J Alzheimer’s Dis JAD (2018) 63:1337–46. 10.3233/JAD-180176 29758946

[29] Beal MF Matson WR Swartz KJ Gamache PH Bird ED. Kynurenine pathway measurements in Huntington’s disease striatum: evidence for reduced formation of kynurenic acid. J Neurochem (1990) 55:1327–39. 10.1111/j.1471-4159.1990.tb03143.x 2144582

[30] Verwaest KA Vu TN Laukens K Clemens LE Nguyen HP Van Gasse B. (1)H NMR based metabolomics of CSF and blood serum: a metabolic profile for a transgenic rat model of Huntington disease. Biochim Biophys Acta (2011) 1812:1371–9. 10.1016/j.bbadis.2011.08.001 21867751

[31] Landwehrmeyer GB. Protocol CS. Enroll-HD Protocol Final Version 1.0 09 September 2011. (2011). pp. 1–64. Available at: https://www.enroll-hd.org/enrollhd_documents/Enroll-HDProtocol-1.0.pdf.

[32] Kieburtz K Penney JB Como P Ranen N Shoulson I Feign A. Unified Huntington’s Disease Rating Scale: reliability and consistency. Huntington Study Group. Mov Disord (1996) 11:136–42. 10.1002/mds.870110204 8684382

[33] Shoulson I Fahn S. Huntington disease: clinical care and evaluation. Neurology (1979) 29:1–3. 10.1212/wnl.29.1.1 154626

[34] Lee JH Lee KU Lee DY Kim KW Jhoo JH Kim JH. Development of the Korean version of the Consortium to Establish a Registry for Alzheimer’s Disease Assessment Packet (CERAD-K): clinical and neuropsychological assessment batteries. J Gerontol Ser B psychol Sci Soc Sci (2002) 57:P47–53. 10.1093/geronb/57.1.p47 11773223

[35] Wechsler D. Manual for the Wechsler Adult Intelligence Scale - Revised. New York: Psychological Corporation (1981).

[36] Stroop RJ. Studies of interference in serial verbal reactions (Reprinted from Journal Experimental-Psychology, Vol 18, Pg 643-662, 1935). J Exp Psychol Gen (1992) 121:15–23. 10.1037/0096-3445.121.1.15

[37] Folstein MF Folstein SE McHugh PR. “Mini-mental state”: A practical method for grading the cognitive state of patients for the clinician. J Psychiatr Res (1975). 10.1016/0022-3956(75)90026-6 1202204

[38] Beck AT Steer RA Brown GK. Beck Depression Inventory-second edition. Manual. Psihologijski mjerni instrumenti - (1996) 93:21. 10.1007/978-3-642-70486-4_13

[39] Caporaso JG Kuczynski J Stombaugh J Bittinger K Bushman FD Costello EK. QIIME allows analysis of high-throughput community sequencing data. Nat Methods (2010) 7:335–6. 10.1038/nmeth.f.303 PMC3156573 20383131

[40] Wasser CI Mercieca EC Kong G Hannan AJ McKeown SJ Glikmann-Johnston Y. Gut dysbiosis in Huntington’s disease: associations among gut microbiota, cognitive performance and clinical outcomes. Brain Commun (2020) 2:fcaa110. 10.1093/braincomms/fcaa110 33005892 PMC7519724

[41] Escobar JS Klotz B Valdes BE Agudelo GM. The gut microbiota of Colombians differs from that of Americans, Europeans and Asians. BMC Microbiol (2014) 14:311. 10.1186/s12866-014-0311-6 25495462 PMC4275940

[42] Finegold SM Dowd SE Gontcharova V Liu C Henley KE Wolcott RD. Pyrosequencing study of fecal microflora of autistic and control children. Anaerobe (2010) 16:444–53. 10.1016/j.anaerobe.2010.06.008 20603222

[43] Barnat M Capizzi M Aparicio E Boluda S Wennagel D Kacher R. Huntington’s disease alters human neurodevelopment. Science (2020) 369:787–93. 10.1126/science.aax3338 PMC7859879 32675289

[44] Alam MT Amos GCA Murphy ARJ Murch S Wellington EMH Arasaradnam RP. Microbial imbalance in inflammatory bowel disease patients at different taxonomic levels. Gut Pathog (2020) 12:1. 10.1186/s13099-019-0341-6 31911822 PMC6942256

[45] Hashimoto Y Hamaguchi M Kaji A Sakai R Osaka T Inoue R. Intake of sucrose affects gut dysbiosis in patients with type 2 diabetes. J Diabetes Invest (2020) 11:1623–34. 10.1111/jdi.13293 PMC7610116 32412684

[46] Plaza-Diaz J Gomez-Fernandez A Chueca N Torre-Aguilar MJ Gil A Perez-Navero JL. Autism Spectrum Disorder (ASD) with and without Mental Regression is Associated with Changes in the Fecal Microbiota. Nutrients (2019) 11:337. 10.3390/nu11020337 PMC6412819 30764497

[47] Lai WT Deng WF Xu SX Zhao J Xu D Liu YH. Shotgun metagenomics reveals both taxonomic and tryptophan pathway differences of gut microbiota in major depressive disorder patients. Psychol Med (2019) 51:90–101. 10.1017/S0033291719003027 31685046

[48] Yang C Qu Y Fujita Y Ren Q Ma M Dong C. Possible role of the gut microbiota-brain axis in the antidepressant effects of (R)-ketamine in a social defeat stress model. Trans Psychiatry (2017) 7:1294. 10.1038/s41398-017-0031-4 PMC5802627 29249803

[49] Berry D Reinisch W. Intestinal microbiota: a source of novel biomarkers in inflammatory bowel diseases? Best practice & research. Clin Gastroenterol (2013) 27:47–58. 10.1016/j.bpg.2013.03.005 23768552

[50] Ijssennagger N van der Meer R van Mil SWC. Sulfide as a Mucus Barrier-Breaker in Inflammatory Bowel Disease? Trends Mol Med (2016) 22:190–9. 10.1016/j.molmed.2016.01.002 26852376

[51] Zhuang P Zhang Y Shou Q Li H Zhu Y He L. Eicosapentaenoic and Docosahexaenoic Acids Differentially Alter Gut Microbiome and Reverse High-Fat Diet-Induced Insulin Resistance. Mol Nutr Food Res (2020) 64:e1900946. 10.1002/mnfr.201900946 32298529

[52] Cai W Xu J Li G Liu T Guo X Wang H. Ethanol extract of propolis prevents high-fat diet-induced insulin resistance and obesity in association with modulation of gut microbiota in mice. Food Res Int (2020) 130:108939. 10.1016/j.foodres.2019.108939 32156386

[53] Paul WE Zhu J. How are T(H)2-type immune responses initiated and amplified? Nat Rev Immunol (2010) 10:225–35. 10.1038/nri2735 PMC3496776 20336151

[54] Guilloteau P Martin L Eeckhaut V Ducatelle R Zabielski R Van Immerseel F. From the gut to the peripheral tissues: the multiple effects of butyrate. Nutr Res Rev (2010) 23:366–84. 10.1017/S0954422410000247 20937167

[55] Feng Z Long W Hao B Ding D Ma X Zhao L. A human stool-derived Bilophila wadsworthia strain caused systemic inflammation in specific-pathogen-free mice. Gut Pathog (2017) 9:59. 10.1186/s13099-017-0208-7 29090023 PMC5657053

[56] Devkota S Wang Y Musch MW Leone V Fehlner-Peach H Nadimpalli A. Dietary-fat-induced taurocholic acid promotes pathobiont expansion and colitis in Il10-/- mice. Nature (2012) 487:104–8. 10.1038/nature11225 PMC3393783 22722865

[57] Lv LJ Li SH Li SC Zhong ZC Duan HL Tian C. Early-Onset Preeclampsia Is Associated With Gut Microbial Alterations in Antepartum and Postpartum Women. Front Cell Infect Microbiol (2019) 9:224:224. 10.3389/fcimb.2019.00224 31297341 PMC6608563

[58] Li F Han Y Cai X Gu M Sun J Qi C. Dietary resveratrol attenuated colitis and modulated gut microbiota in dextran sulfate sodium-treated mice. Food Funct (2020) 11:1063–73. 10.1039/c9fo01519a PMC7122795 31825043

[59] Liu G Bei J Liang L Yu G Li L Li Q. Stachyose Improves Inflammation through Modulating Gut Microbiota of High-Fat Diet/Streptozotocin-Induced Type 2 Diabetes in Rats. Mol Nutr Food Res (2018) 62:e1700954. 10.1002/mnfr.201700954 29341443

[60] Gibson GR Roberfroid MB. Dietary modulation of the human colonic microbiota: introducing the concept of prebiotics. J Nutr (1995) 125:1401–12. 10.1093/jn/125.6.1401 7782892

[61] Karlsson FH Tremaroli V Nookaew I Bergstrom G Behre CJ Fagerberg B. Gut metagenome in European women with normal, impaired and diabetic glucose control. Nature (2013) 498:99–103. 10.1038/nature12198 23719380

[62] Strati F Cavalieri D Albanese D De Felice C Donati C Hayek J. New evidences on the altered gut microbiota in autism spectrum disorders. Microbiome (2017) 5:24. 10.1186/s40168-017-0242-1 28222761 PMC5320696

[63] Tomova A Husarova V Lakatosova S Bakos J Vlkova B Babinska K. Gastrointestinal microbiota in children with autism in Slovakia. Physiol Behav (2015) 138:179–87. 10.1016/j.physbeh.2014.10.033 25446201

[64] Wang W Chen L Zhou R Wang X Song L Huang S. Increased proportions of Bifidobacterium and the Lactobacillus group and loss of butyrate-producing bacteria in inflammatory bowel disease. J Clin Microbiol (2014) 52:398–406. 10.1128/JCM.01500-13 24478468 PMC3911339

[65] Breban M Tap J Leboime A Said-Nahal R Langella P Chiocchia G. Faecal microbiota study reveals specific dysbiosis in spondyloarthritis. Ann Rheum Dis (2017) 76:1614–22. 10.1136/annrheumdis-2016-211064 28606969

[66] Picchianti-Diamanti A Panebianco C Salemi S Sorgi ML Di Rosa R Tropea A. Analysis of Gut Microbiota in Rheumatoid Arthritis Patients: Disease-Related Dysbiosis and Modifications Induced by Etanercept. Int J Mol Sci (2018) 19:2938. 10.3390/ijms19102938 PMC6213034 30261687

[67] Rahnavard G Franzosa EA McIver LJ Schwager E Lloyd-Price J Weingart G. High-sensitivity pattern discovery in large multi"omic datasets. Harvard T.H. Chan School of Public Health (2019). Available at: https://huttenhower.sph.harvard.edu/halla.

