# Supplementary material for: Altered Gut Microbiota Related to Inflammatory Responses in Patients With Huntington’s Disease
Source: Front Immunol. 2021 Feb 19;11:603594. doi: 10.3389/fimmu.2020.603594 (PMC7933529; doi:10.3389/fimmu.2020.603594)
Supplement: Supplementary file 1 [file Table_1.docx]

**Supplementary Table 1 |** **Demographics and clinical characteristics of manifest and premanifest HD patients**

|  | Manifest HD group | Premanifest HD group | *p* value |
| --- | --- | --- | --- |
| N | 24 | 9 | NA |
| M:F | 11:13 | 4:5 | 0.943^+^ |
| Age (y/o) | 46.0 (12.8) ^a^ | 33.3 (6.5) ^a^ | 0.0083* |
| BMI (kg/m^2^) | 20.6 (3.6) ^a^ | 22.9 (3.6) ^a^ | 0.1326* |
| CAG repeat number | 42.0 (3.5) ^b^ | 42.0 (4.0) ^b^ | 0.536^#^ |
| UHDRS-M | 53.5 (33.4) ^a^ | 0 (1.0) ^b^ | < 0.001^#^ |
| FAS scores | 19.0 (11.3) ^b^ | 23.0(0) ^b^ | 0.001^#^ |
| TFC scores | 8.5 (10.0) ^b^ | 13.0(0) ^b^ | < 0.001^#^ |
| SIT scores | 17.1 (6.6) ^a^ | 39.0 (15.3) ^a^ | < 0.001* |
| SDMT scores | 19.2 (9.9) ^a^ | 40.0(16.0) ^b^ | < 0.001^#^ |
| CFT scores | 10.0 (6.0) ^b^ | 19.2(6.1)^a^ | 0.005^#^ |
| BDI-II scores | 11.8 (11.5) ^a^ | 1.0(7.0) ^b^ | 0.071^#^ |
| MMSE scores | 27.0 (3.3) ^b^ | 30.0(0) ^b^ | < 0.001^#^ |

*Unpaired t-test; ^#^Mann-Whitney U test; ^+^Pearson's Chi-squared test; the data are presented as the mean (SD)^a^ or median (IQR)^b^; HD = Huntington's disease; NA = not applicable; SD = standard deviation; IQR = interquartile range; M:F = male: female; y = years; y/o = years old; BMI = body mass index; UHDRS-M = motor section of Unified Huntington’s Disease Rating Scale; FAS = Functional Assessment Scale; TFC = Total Functional Capacity; SIT = Stroop Interference Test; SDMT = Symbol Digit Modalities Test; CFT = Category Fluency Test; BDI-II = Beck Depression Inventory II; MMSE = Mini-Mental State Examination.
