# Supplementary figures and images for: Altered Gut Microbiota Related to Inflammatory Responses in Patients With Huntington’s Disease
Source: Front Immunol. 2021 Feb 19;11:603594. doi: 10.3389/fimmu.2020.603594 (PMC7933529; doi:10.3389/fimmu.2020.603594)

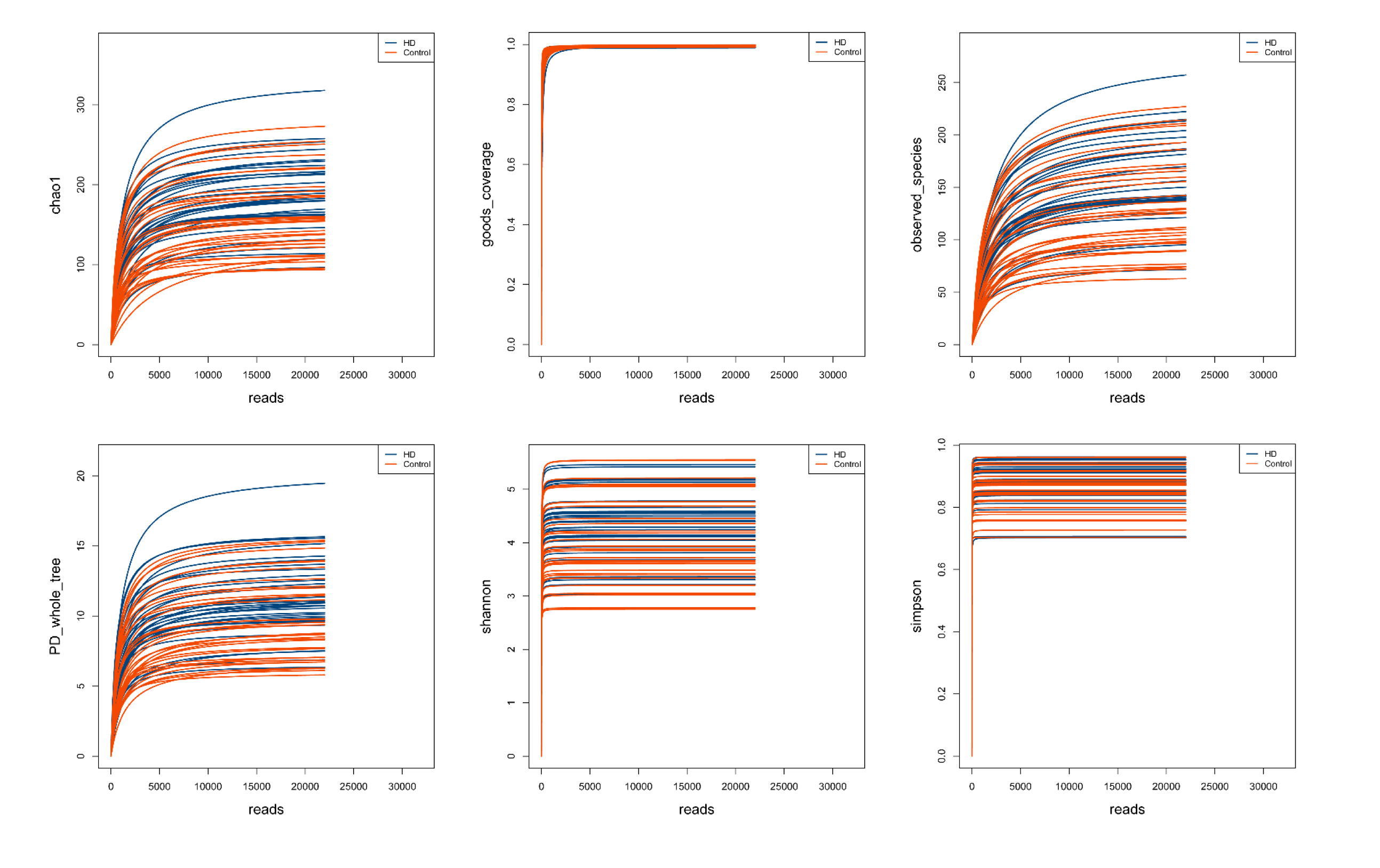

Supplement: Supplementary Figure 1 — Dilution curves of α-diversity index. As the amount of sequencing increased, more species were found, and no new OTU was found by increasing the number of sampling strips until the species was saturated. [file Image_1.tif]

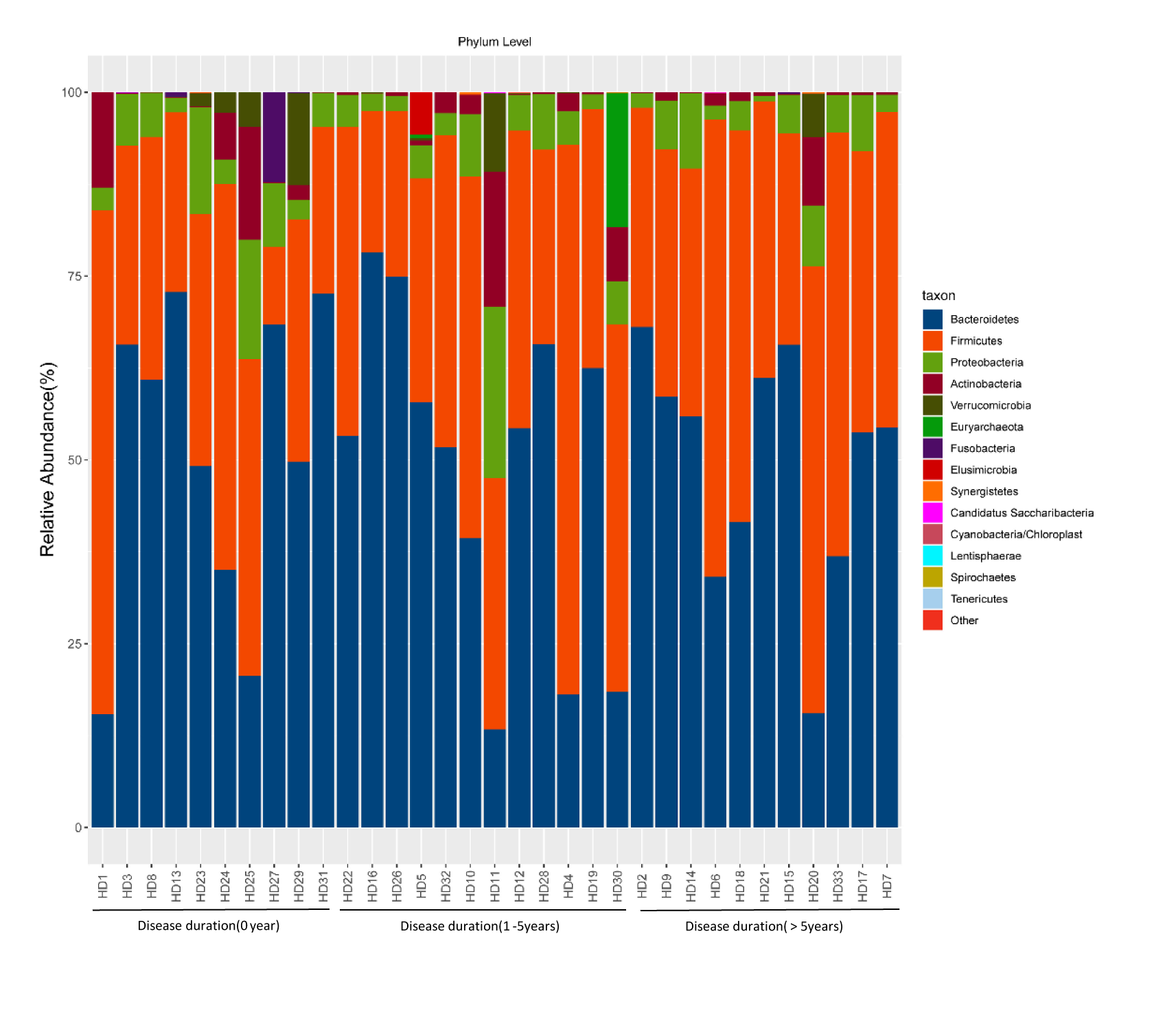

Supplement: Supplementary Figure 2 — Species profiling histogram of the HD samples according to HD duration at phylum classification level. [file Image_2.tif]

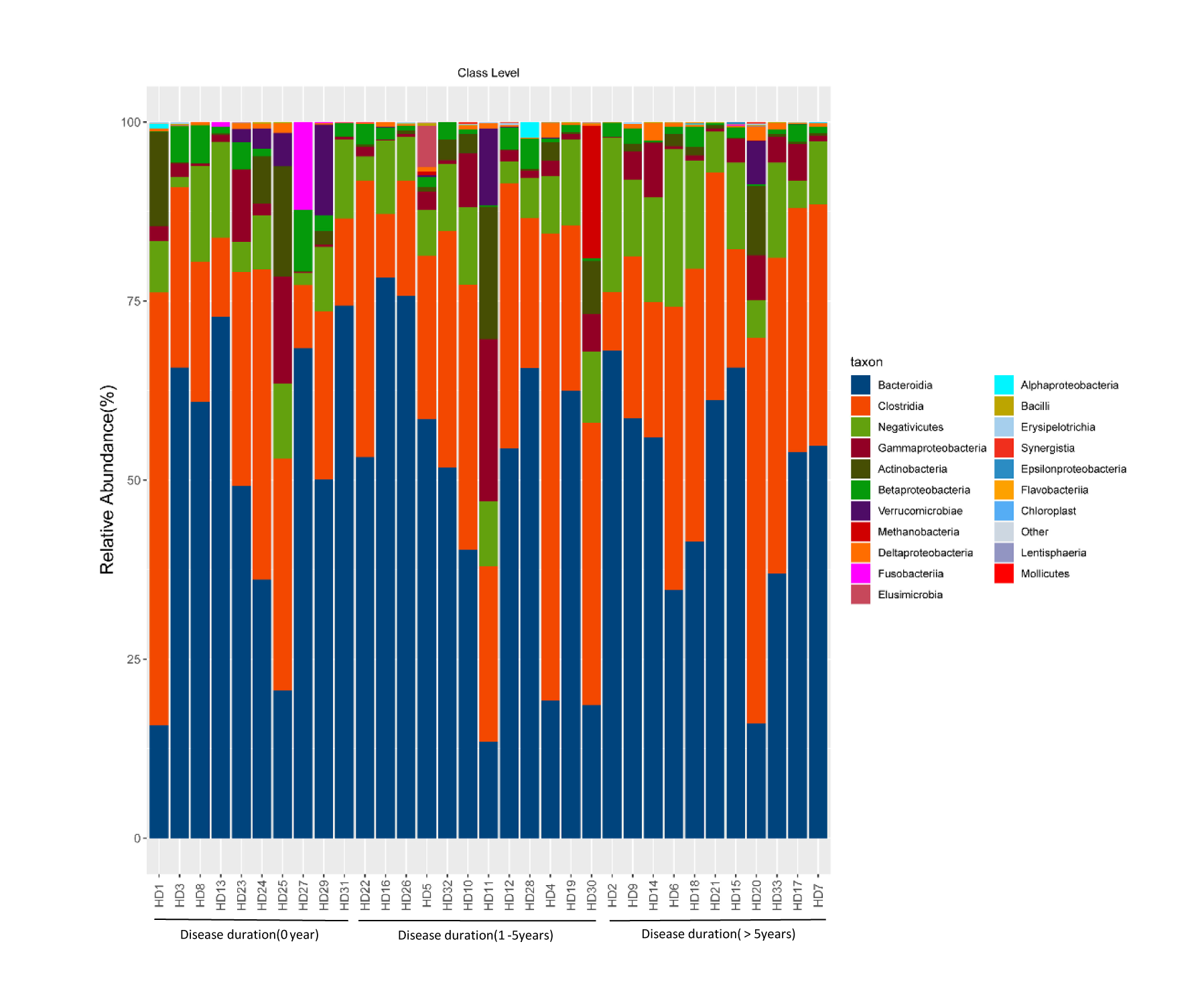

Supplement: Supplementary Figure 3 — Species profiling histogram of the HD samples according to HD duration at class classification level. [file Image_3.tif]

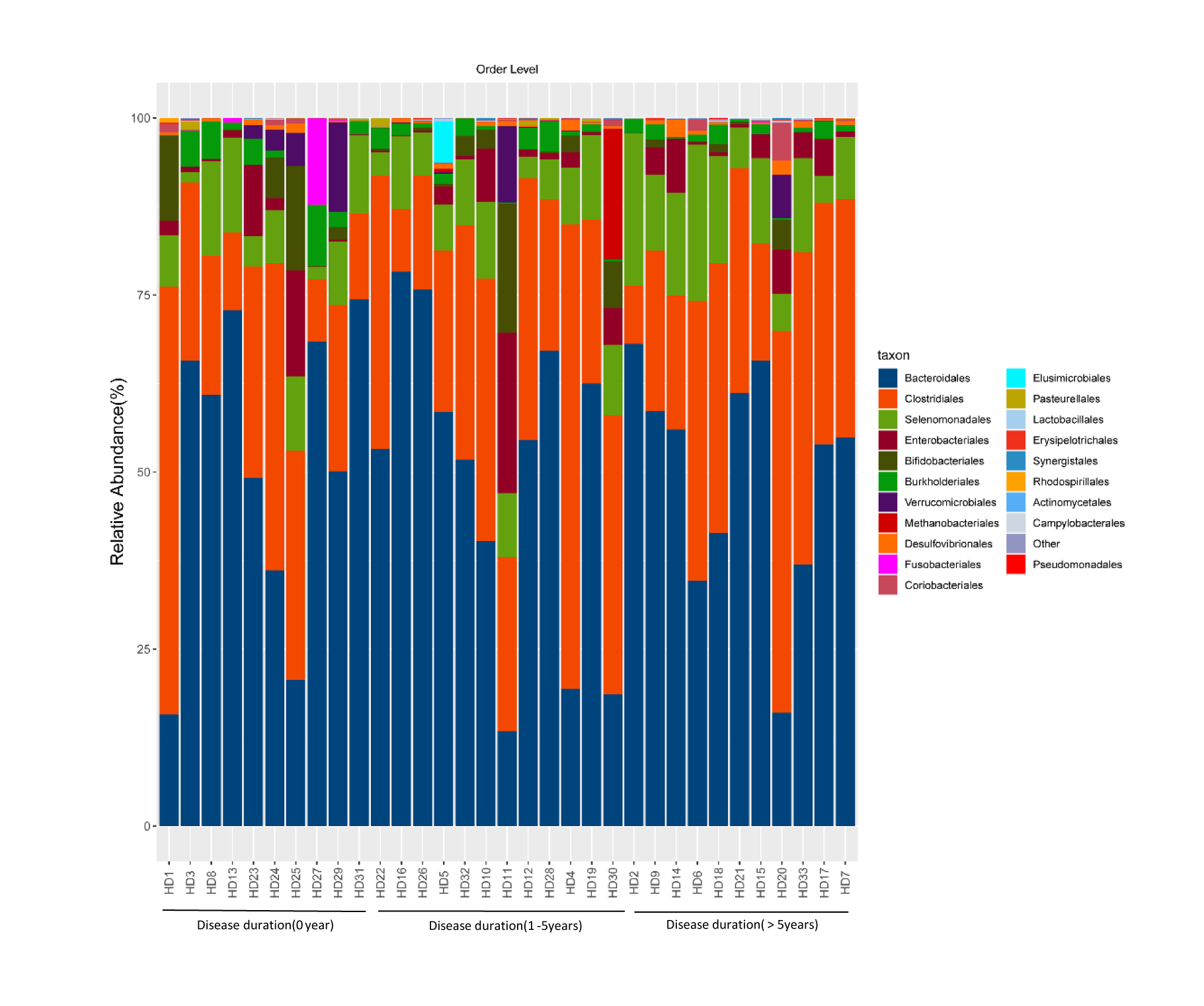

Supplement: Supplementary Figure 4 — Species profiling histogram of the HD samples according to HD duration at order classification level. [file Image_4.tif]

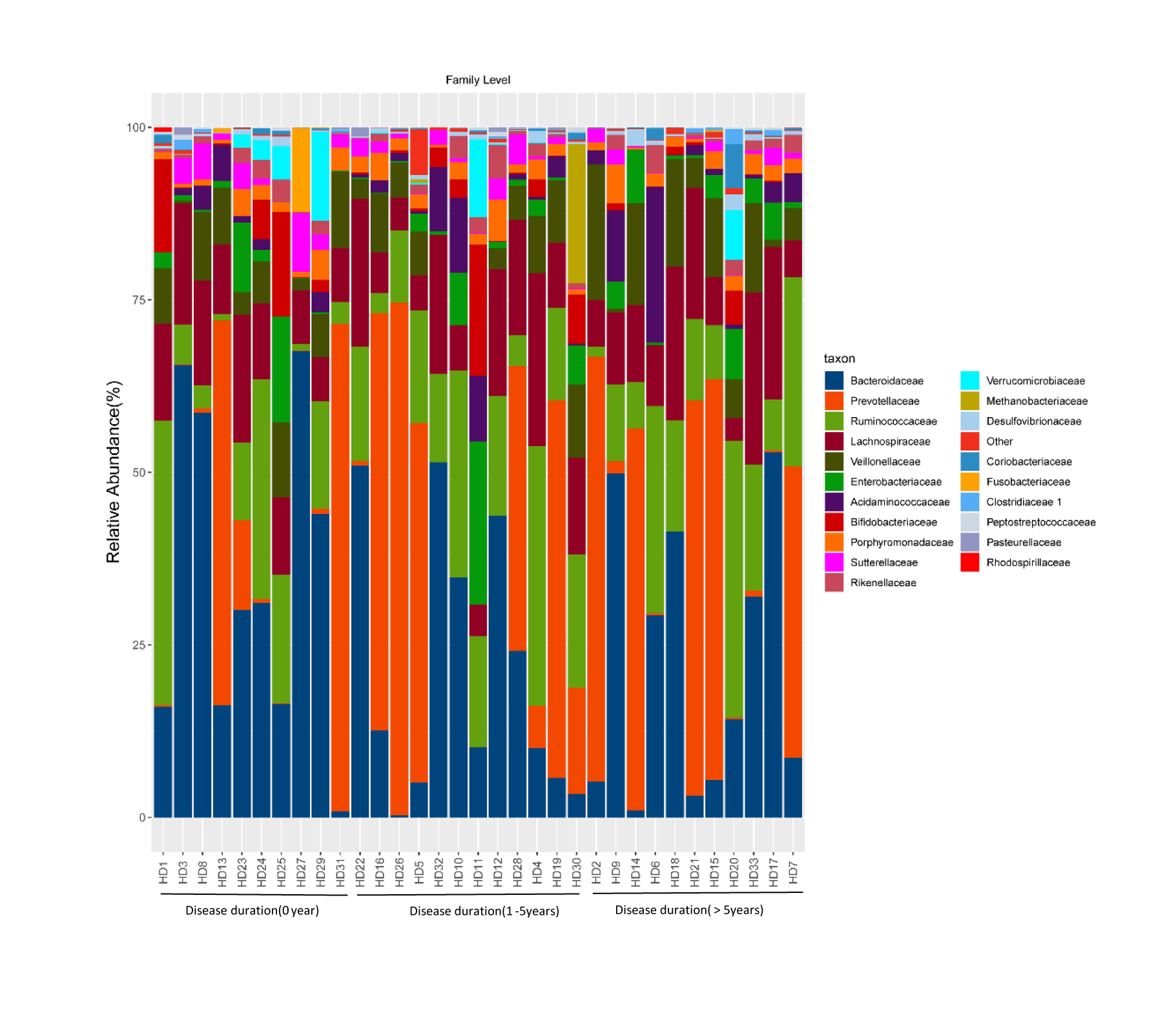

Supplement: Supplementary Figure 5 — Species profiling histogram of the HD samples according to HD duration at family classification level. [file Image_5.tif]

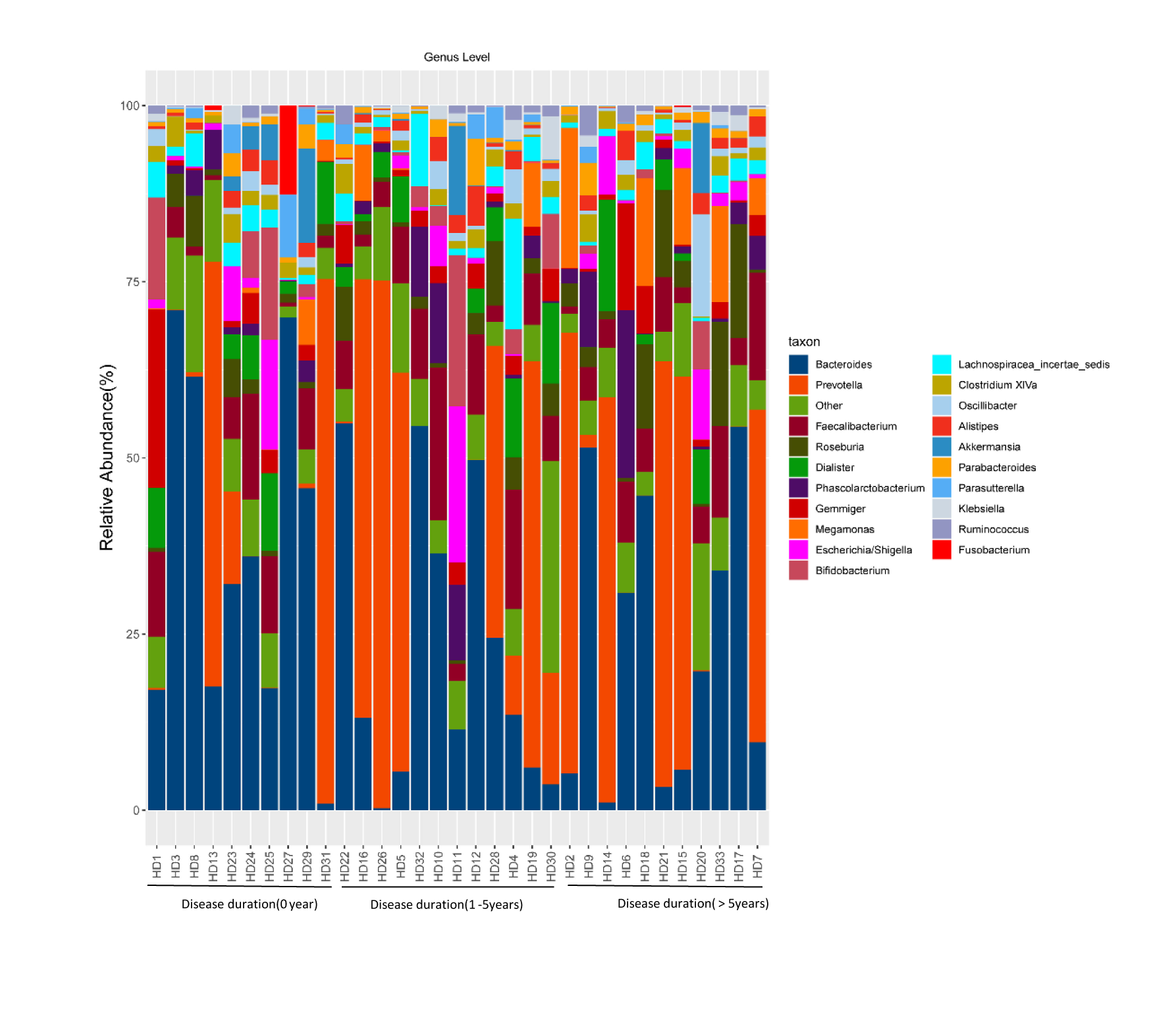

Supplement: Supplementary Figure 6 — Species profiling histogram of the HD samples according to HD duration at genus classification level. [file Image_6.tif]
